# Apelin and vascular endothelial growth factor are associated with mobilization of endothelial progenitor cells after acute myocardial infarction

**DOI:** 10.7555/JBR.26.20120052

**Published:** 2012-10-20

**Authors:** Jiaxin Ye, Ping Ni, Lina Kang, Biao Xu

**Affiliations:** a Department of Cardiology, Drum Tower Clinical College Affiliated with Nanjing Medical University, Nanjing, Jiangsu 210008, China;; b Department of Cardiology, Drum Tower Hospital (L.N.K.), Nanjing, Jiangsu 210008, China.

**Keywords:** apelin, vascular endothelial growth factor (VEGF), stromal cell-derived growth factor-1 (SDF-1), endothelial progenitor cells (EPCs)

## Abstract

This study was designed to determine the levels of early endothelial progenitor cells (EPCs), apelin, vascular endothelial growth factor (VEGF) and stromal cell-derived growth factor-1 (SDF-1) after acute myocardial infarction (AMI), and to investigate the relationships between these cytokines and early EPCs. Early EPCs, defined as CD133^+^, KDR^+^, and CD34^+^ cells, were quantified by flow cytometry. The levels of early EPCs and those cytokines in AMI patients were significantly different from those with coronary artery disease or controls (*P* < 0.05). Plasma apelin levels were inversely correlated with Gensini score and early EPCs (both *P* < 0.01). Early EPCs, VEGF and SDF-1 showed different patterns of changes in AMI patients during the first 24 h. The trend in the change of early EPCs was proportionally correlated with that of VEGF (*P* < 0.05). AMI patients exhibited increased early EPCs with remarkably decreased apelin levels and enhanced VEGF levels.

## INTRODUCTION

Endothelial progenitor cells (EPCs) are circulating angioblasts derived from human bone marrow with a potential to differentiate into endothelial cells[1],[2]. Circulating EPCs are defined as cells positive for haematopoietic stem cell markers, such as CD34, and endothelial markers, such as vascular endothelial growth factor (VEGFR) 2[3]. Early EPCs in the bone marrow, or immediately after their migration into the systemic circulation, are positive for CD133/ CD34/ VEGFR2 (KDR), whereas circulating EPCs are positive for CD34/ VEGFR2/ CD31/ VE-cadherin. They obviously lose CD133 and begin to express von Willebrand factor[4]. Increased EPCs in the peripheral blood have been reported in various pathologic conditions involving severe endothelial perturbation, including inflammatory disease, acute myocardial infarction (AMI), unstable angina (UA), and critical limb ischemia[5]–[7]. The elevation of early EPCs in peripheral blood represents the ability to assess endothelial damage. Early EPCs possess the capacity to home to sites of vascular injury, to produce neoangiogenesis in vivo and to maintain the homeostasis of vascular endothelia[8]. In some pathologic conditions, various cytokines, particularly vascular endothelial growth factor (VEGF), stromal cell-derived growth factor-1 (SDF-1), granulocyte colony stimulating factor (G-CSF), and interleukin-8 (IL-8)[9]–[11] have been shown to have beneficial effects on the mobilization of EPCs, by increasing their number and functionality[12]–[16].

Apelin is a peptide which appears to act as an endogenous ligand for the G-protein-coupled angiotensin II protein J (APJ) receptor that is expressed at the surface of some cell types[17]. In the vascular system, APJ and apelin are known to be expressed in endothelia and vascular smooth muscle cells[18]. In vitro and in vivo studies have demonstrated that, apelin is upregulated in response to hypoxia in peripheral and cardiac tissues[19],[20]. In rat experimental AMI, an increase in apelin and APJ expression was observed[21]. Apelin/APJ deficiency is preventative against oxidative stress-linked atherosclerosis, and the apelin/APJ system works as a mediator of oxidative stress in vascular tissue[22]. Activation of apelin, like VEGF[23], promotes the formation of new blood vessels and the proliferation of endothelial cells, umbilical endothelial cells and gastric cells. Thus, we hypothesized that a correlation may exist between early EPCs and apelin. We performed this study to investigate the mobilization of early EPCs marked as CD45^low/−^ CD34^+^KDR^+^CD133^+^ and evaluate the correlation of cytokines with the level of early EPCs during the acute phase of cardiac disease.

## SUBJECTS AND METHODS

### Patients

The protocol was approved by the Institutional Ethics Committee in accordance with the principles outlined in the Declaration of Helsinki, with informed consent provided by each patient before enrollment. This was an observational study, and none of the participants could influence clinical practice. A total of 159 patients, who underwent diagnostic angiography, and some of whom received percutaneous coronary intervention (PCI) (including percutaneous transluminal coronary angioplasty), were enrolled at a single facility between June 2010 and March 2011. Control subjects (Control) were defined as those having angiographically normal coronary arteries. Coronary artery disease (CAD) patients enrolled should meet the following criteria: (1) history of transient episodes of typical exertional chest pain which remains unchanged for more than 3 months before blood sample collection (excluding rest angina); (2) at least one coronary stenosis producing ≥50% diameter reduction. AMI patients (AMI) enrolled were defined as those with symptom onset within the past 24 h. The diagnostic criteria of AMI were defined as follows: (1) creatine kinase MB (CK MB) fraction elevated above twice the upper normal limit with raised troponin T (TNT) levels; (2) concurrent prolonged chest pain or discomfort; (3) persistent ST-segment or T-wave changes or with presumed new left bundle-branch block. All AMI patients received a reasonable loading clopidogrel and aspirin on admission. Further analysis was given to AMI patients on the trend of EPCs and plasma concentration of cytokines during the first 24 h after admission. All patients received proper therapy according to their syndromes and diseases. Inclusion criteria included the ability to give informed consent and referral for coronary angiography for the evaluation of CAD. Exclusion criteria included cardiogenic shock, AMI in preceding years, malignancies, diabetes mellitus, current smoking, acute or chronic inflammatory diseases, infection with human immunodeficiency virus, and treatment with immunosuppressive medications including steroids, pregnancy, or any hematological disorders, and history of blood-transfusion within the past 2 weeks. Patients with thrombocytopenia (< 100×10^3^ cells/mm^3^), anemia (Hb < 10 mg/dL) or renal failure (Cr > 2.5 mg/dL), were also excluded from the study.

### Angiographic assessment

Standardized angiographic projections that showed coronary artery stenosis at its highest severity were recorded in every patient. Coronary angiograms were interpreted by two independent cardiologists. Percentage of coronary narrowing was calculated by comparison of the minimum diameter of the segment with the diameter of an adjacent angiographically normal coronary segment. Gensini score, which considers both the extent and the severity of the lesions at coronary angiography, was calculated for each patient and used for the assessment of coronary narrowing severity[24]. Gensini score system also grades the stenosis in the epicardial coronary arteries (1 for 1%-25% stenosis, 2 for 26%-50% stenosis, 4 for 51%-75% stenosis, 8 for 76%-90% stenosis, 16 for 91%-99% stenosis, and 32 for total occlusion), which is then multiplied by a constant number determined according to the anatomical position of the lesion.

### Study protocol

Peripheral blood samples were collected on admission of all patients (baseline), and were also collected for AMI patients receiving primary PCI before the procedure. All stents used were rapamycin-eluting stents. For patients who received primary PCI according to guidelines, samples were drawn at 3, 6, 12, and 24 h after successful recanalization of the occluded infarct-related coronary artery (post-PCI 3 h, post-PCI 6 h, post-PCI 12 h, and post-PCI 24 h), respectively. For patients who did not receive primary PCI, peripheral blood samples were collected at 3, 6, 12, and 24 h after admission (non-PCI 3 h, non-PCI 6 h, non-PCI 12 h, and non-PCI 24 h), respectively.

### Collection of blood samples

Peripheral blood samples were collected into a 4-mL Vacutainer (Becton Dickinson, Basel, Switzerland) tube containing liquid EDTA as an anticoagulant. Plasma was separated by prompt centrifugation (Histopaque-1077, Sigma-Aldrich, St. Louis, MO, USA) at 400 *g* at 4°C for 10 min and then stored at -80°C immediately. All procedures including EPC isolation, flow cytometric analysis and blood plasma centrifugation were performed within 2 h after blood collection.

### Monoclonal antibodies

The following directly conjugated mouse anti-human monoclonal antibodies were used for flow cytometry: fluorescein isothiocyanate (FITC)-labeled anti-CD34 (eBioscience, San Diego, CA, USA), peridinim chlorophyll (PerCP)-cy5.5-labeled anti-CD45 (eBioscience), allophycocianin (APC) -labeled anti-VEGFR2 (KDR) (R&D Systems, Minneapolis, MN, USA), and phycoerthrin (PE)-labeled anti-CD133 (Miltenyi Biotec, Auburn, MN, USA).

### Multicolor staining and flow analysis

Mononuclear cells were isolated from 1 mL peripheral blood with the use of a Ficoll density gradient (Biocoll, Biochrom, USA) by a density-gradient centrifugation (Histopaque-1077, Sigma-Aldrich) according to standard protocols, and then cells were centrifuged and resuspended in 1 mL phosphate-buffered saline (PBS), pH 7.2. A panel of monoclonal antibodies including anti-CD45 (leukocyte common antigen; to exclude haematopoietic cells), anti-CD34 (marker for bone marrow-derived progenitor stem cells), CD133 (immature haematopoietic stem cell marker), KDR (endothelial marker) and appropriate analysis gates were used to enumerate EPCs. To quantify the content of CD45^low/−^CD34^+^ KDR^+^CD133^+^cells, 100 µL of sample was incubated for 20 min at 4°C in the dark with 5 µL of PerCP-cy5.5-CD45 and FITC-CD34, 10 µL of PE-CD133 and APC-KDR. Cells labeled with PE-, FITC-, PerCP-cy5.5 and APC-conjugated isotypic monoclonal antibodies were used as controls to determine the background of fluorescence. After staining, red blood cell components in cells were lysed with 2 mL lysing solution (Biolegend, San Diego, CA, USA) according to the manufacturer's instruction. The remaining cells were washed in 1 mL PBS (300 g, 5 min) and finally resuspended in 500 µL PBS. Then, analysis was performed within 30 min. Nucleated cells from specimens were evaluated using a FACS Canto flow cytometer (BD Biosciences CA) as described previously[25], with identical set-up parameters between samples. The data was analyzed using BD FACSDiva software. All samples were measured in duplicate. For fluorescence-activated cell sorting analysis, 1×10^6^ mononuclear cells were acquired and scored. Samples were subjected to a 2D side scatter-fluorescence dot plot analysis. The same trained operator blinded to this study performed all the tests.

### Plasma concentration of cytokines

The plasma levels of apelin, VEGF, and SDF-1 were respectively determined in duplicate using commercially available ELISA kits according to the manufacturers' guidelines (Apelin: Phoenix Pharmaceuticals, Belmont, CA, USA; VEGF and SDF-1: R&D Systems).

### Statistical analysis

Data were presented as mean±standard deviation (SD). The numbers of Gensini were considered as a continuous variable. Normally distributed variable were compared by means of independent *t* test. Correlations were assessed with linear regression analysis and Spearman's rank correlation test. All tests were two-sided, and statistical significance was considered for *P* values < 0.05. All statistical analyses were performed using SPSS version 17.0 for Windows (Chicago, IL, USA).

## RESULTS

### Baseline characteristics

This study included 87 consecutive patients with AMI (67 men and 20 women; age 69±13 years). Thirty-three patients with angiographically documented stable CAD (24 men and 9 women, age 64±10 years) and 39 control subjects (30 men and 9 women, age 66±8 years) were enrolled in the study. The patients' baseline demographic characteristics are shown in ***Table 1***. There were no differences in age, body mass index (BMI), cardiovascular risk factors, and current medications (β-adrenoceptor blockers, calcium channel blockers, and angiotensin-converting enzyme inhibitors or angiotensin receptor blockers) among the control, CAD and AMI groups (all *P* > 0.05). Additionally, there were no differences in age, infarct-related coronary vessel, NYHA-classification, hemoglobin, and red blood cells among these groups (all *P* > 0.05).

### Early EPCs and plasma levels of apelin, VEGF and SDF-1 at baseline

**Fig. 1:**
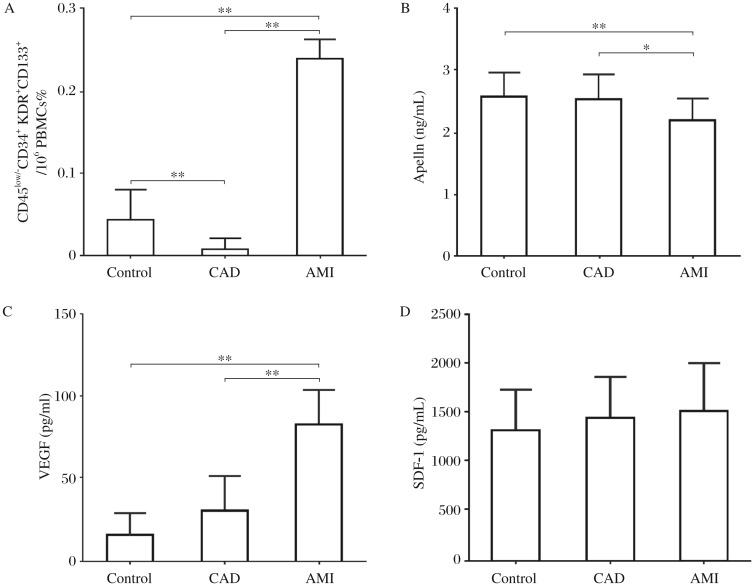
Early EPCs and plasma apelin, VEGF and SDF-1 at baseline. A: Early EPCs marked as CD45^low/−^CD34^+^ KDR^+^CD133^+^ were measured by flow cytometry. B, C and D: the level of apelin, VEGF and SDF-1 were determined using ELISA kits. **P < 0.01, *P < 0.05. Results are represented as mean±SD. AMI: acute myocardial infarction; CAD: coronary artery disease. PBMCs: peripheral blood mononuclear cells. SDF-1: stromal cell-derived factor-1; VEGF: vascular endothelial growth factor. AMI *n* = 87; Controls *n* = 39; CAD *n* = 33.

The number of CD45^low/−^CD34^+^ KDR^+^CD133^+^ early EPCs in AMI patients was 6-fold higher than that of controls, and 24-fold higher than that of CAD patients (Control vs CAD/AMI, 0.04%±0.03% vs 0.01%±0.01%/0.24%±0.02%, *P*_Control vs CAD_ < 0.01, *P*_Control vs AMI_ < 0.01, *P*_CAD vs AMI_ < 0.01) (***Fig. 1A***). Plasma apelin levels in AMI patients were 0.85-fold lower than those of controls, and 0.87-fold lower than those of CAD patients (Control vs CAD/AMI, 2.60±0.36 vs 2.54±0.47/2.21±0.34 ng/mL, *P*_Control vs CAD_ > 0.05, *P*_Control vs AMI_ < 0.01, *P*_CAD vs AMI_ < 0.05) (***Fig. 1B***). Plasma VEGF levels in AMI were 5.1-fold higher than those of controls, and 2.8-fold higher than those of CAD patients (Control *vs* CAD/AMI, 16.04±12.05 vs 29.93±27.02/82.58±32.38 pg/mL, *P*_Control vs CAD_ > 0.05, *P*_Control vs AMI_ < 0.01, *P*_CAD vs AMI_ < 0.01) (***Fig. 1C***). There was no difference in plasma SDF-1 levels among AMI, Control and CAD (Control vs CAD/AMI, 1316.27±653.01 vs 1442.47±446.89/1511.59±528.94 pg/mL, *P*_Control vs CAD_ > 0.05, *P*_Control vs AMI_ > 0.05, *P*_CAD vs AMI_ > 0.05) (***Fig. 1D***).

The plasma level of apelin at baseline was inversely correlated with Gensini score (***Table 1***) (*r* = -0.47, *P* < 0.01) and early EPCs levels (%) (*r* = -0.41, *P* < 0.01).

**Table 1:** Baseline characteristics and admission data of the studdy population

Characteristics	AMI (*n* = 87)	CAD (*n* = 33)	Control (*n* = 39)	*P* value
General data				
Age (years)	69±13	64±10	66±8	0.07
Gender (female/male)	20/67	9/24	9/30	0.88
Body mass index (kg/m^2^)	24.08±3.21	24.33±2.47	24.45±2.93	0.88
Systolic blood pressure (mmHg)	137.65±21.14	129.32±17.60	127.41±18.40	0.087
Gensini	067.53±37.24	045.93±29.17	02.57±1.67	*P* < 0.001
Cardiovascular risk factors [*n*(%)]				
Hypercholesterolemia	09 (10.3%)	03 (9.09%)	02 (5.13%)	0.634
Hypertension	70 (80.5%)	29 (87.9%)	33 (84.6%)	0.600
Laboratory parameters				
CK-MB (U/L)	048.71±89.64	12.09±8.74	10.45±2.43	*0.010*
Troponin T (µg/L)	00.35±0.53	00.05±0.00	00.05±0.01	*0.000*
Triglycerides (mmol/L)	01.81±1.20	01.96±1.29	01.52±0.71	0.333
Medication [*n*(%)]				
Beta-blocker	21 (24.1%)	05 (15.2%)	3 (7.7%)	0.077
Calcium channel blockers	25 (28.7%)	11 (33.3%)	05 (12.8%)	0.092
Diuretics	4 (4.6%)	0 (-)000	1 (2.6%)	0.426
Statin	19 (21.8%)	05 (15.2%)	3 (7.7%)	0.142
ACEI or ARB	46 (52.9%)	13 (39.4%)	19 (48.7%)	0.421
Medical history [*n*(%)]				
stroke	4 (4.6%)	1 (3.0%)	0 (-)	0.395
PCI	8 (9.2%)	1 (3.0%)	0 (-)	0.092

AMI: acute myocardial infarction; CAD: coronary artery disease; CK-MB: creatine kinase MB fraction; ACEI: angiotensin-converting enzyme inhibitors; ARB: angiotensin receptor blockers; PCI: percutaneous coronary intervention. The body-mass index is the weight in kilograms divided by the square of the height in meters. Data are presented as number (percentage) or as mean±SD. *P < 0.05.

### Acute changes in early EPCs and plasma VEGF and SDF-1 levels during the first 24 h in AMI

#### CD45^low/–^CD34^+^ KDR^+^CD133^+^EPCs

Since the mobilization of EPCs may be affected by the PCI procedure[26], we investigated early EPCs of AMI in the two groups during the first 24 h. For AMI patients who received primary PCI (PCI, *n* = 43), early EPCs reached the peak at the baseline, and declined in the next 24 h, without significant difference in the levels of early EPCs at baseline and post-PCI 3 h (baseline vs post-PCI 3 h, 0.24%±0.02% vs 0.20%±0.07%, *P*_baseline *vs* post-PCI 3 h_ > 0.05). EPCs at baseline was 3-, 1.5-, and 2.7-folds higher than those at post-PCI 6, 12 and 24 h, respectively (baseline vs post-PCI 6 h/post-PCI 12 h/post-PCI 24 h, 0.24%±0.02% vs 0.08%±0.06%/0.16%±0.06%/0.09%±0.03%, *P*_baseline vs post-PCI 6 h_ < 0.01, *P*_baseline vs post-PCI 12 h_ < 0.01, and *P*_baseline vs post-PCI 24 h_ < 0.01).

**Fig. 2:**
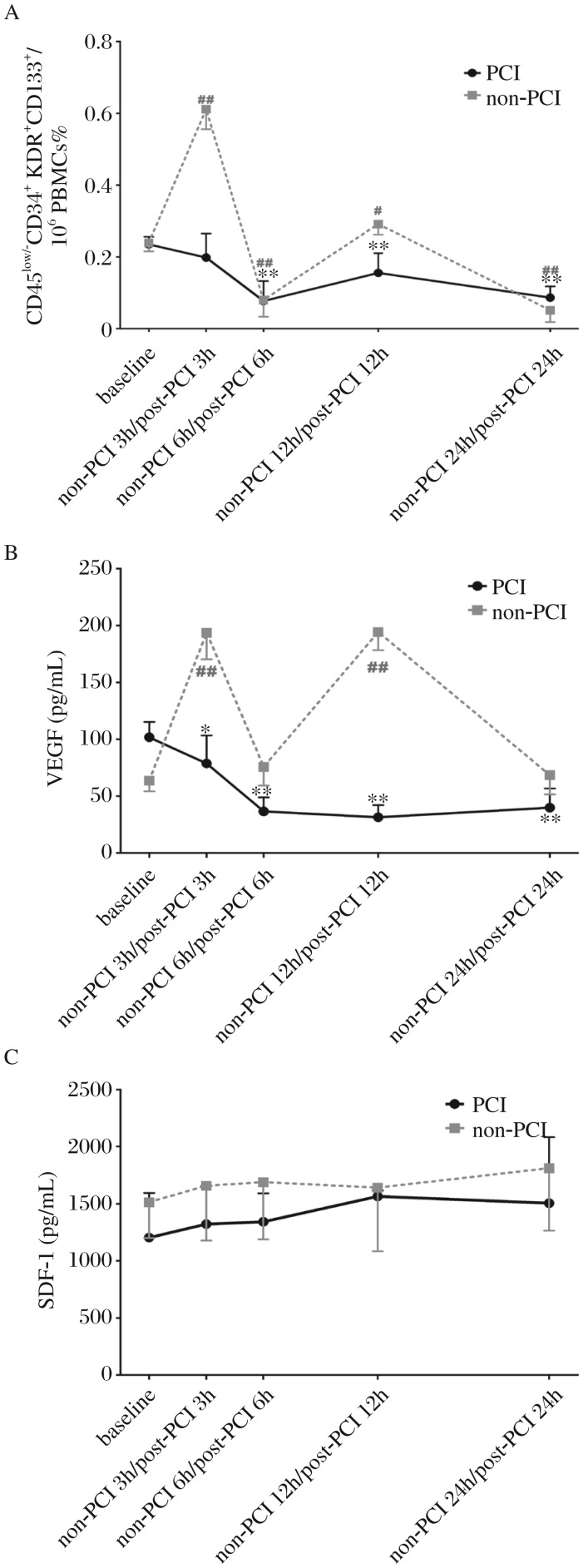
Acute changes of early EPCs and plasma VEGF and SDF-1 levels during the first 24 h in AMI. A: Early EPCs marked as CD45^low/−^CD34^+^ KDR^+^CD133^+^ measured by flow cytometry. B and C: the level of VEGF and SDF-1 was determined using ELISA kits. PBMCs: peripheral blood mononuclear cells. SDF-1: stromal cell-derived factor-1; VEGF: vascular endothelial growth factor. PCI (*n* = 43), non-PCI (*n* = 44). Compared with the baseline of receiving primary PCI (PCI), **P < 0.01, *P < 0.05. Compared with the baseline of not receiving primary PCI (non-PCI), ^##^*P* < 0.01, ^#^*P* < 0.05.

For AMI patients who did not receive primary PCI (non-PCI, *n* = 44), the level of early EPCs (3 h) was maximized during the first 24 h and reached another high spot at 12 h later (baseline vs non-PCI 3 h/non-PCI 12 h, 0.24%±0.02% vs 0.61%±0.06%/0.29%±0.03%, *P*_baseline vs non-PCI 3 h_ < 0.01, *P*_baseline vs non-PCI 12 h_ < 0.05). The early EPCs level at baseline was 3- and 5-folds higher than that at 6 h and 24 h, respectively (baseline vs 6 h/24 h, 0.24%±0.024% vs 0.08%±0.047%/0.052%±0.033%, *P*_baseline vs non-PCI 6 h_ < 0.01, and *P*_baseline vs non-PCI 24 h_ < 0.01) (***Fig. 2A***).

#### Plasma concentrations of cytokines

**Fig. 3:**
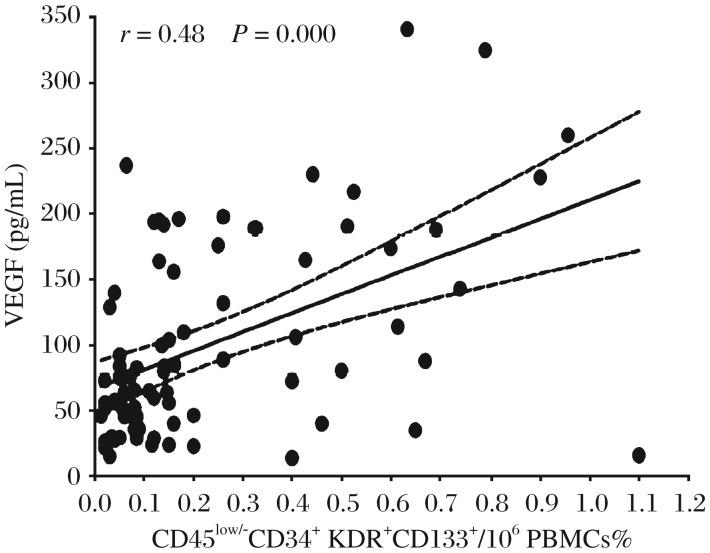
The correlation of early EPCs with VEGF During the first 24 h in AMI. Spearman's correlation coefficients (r) and P value are indicated. Pair of dotted lines near the fitted line is the 95% median confidence limits for the fitted line. VEGF: vascular endothelial growth factor. PBMCs: peripheral blood mononuclear cells. Early EPCs marked as CD45^low/−^CD34^+^ KDR^+^CD133^+^.

The pro-angiogenic factor VEGF level in plasma followed a similar pattern to early EPCs. In the PCI group, VEGF peaked at the baseline and then significantly declined during the next 24 h (baseline vs post-PCI 3 h/post-PCI 6 h/post-PCI 12 h/post-PCI 24 h, 101.69±13.53 vs 78.60±24.64/36.46±12.36/ 31.36±10.71/39.72±16.97 pg/mL, *P*_baseline vs post-PCI 3 h_ < 0.05, *P*_baseline vs post-PCI 6 h_ < 0.01, *P*_baseline vs post-PCI 12 h_ < 0.01, and *P*_baseline vs post-PCI 24 h_ < 0.01). An M-shaped pattern with a significant increase at 3 and 12 h after admission emerged in non-PCI patients (baseline vs 3 h/12 h, 63.63±9.52 vs 193.74±23.46/194.32±15.97 pg/mL, *P*_baseline vs non-PCI 3 h_ < 0.01, *P*_baseline vs non-PCI 12 h_ < 0.01). There was no significant difference in the levels of EPCs at baseline and at non-PCI 6 and 24 h (baseline vs non-PCI 6 h/non-PCI 24 h, 63.63±9.52 vs 75.64±16.53/68.40±16.85 pg/mL, *P*_baseline vs non-PCI 6 h_ > 0.05, *P*_baseline vs non-PCI 24 h_ > 0.05) (***Fig. 2B***). Plasma SDF-1 in the two groups remained at similar levels during the first 24 h (baseline vs post-PCI 3 h/post-PCI 6 h/post-PCI 12 h/post-PCI 24 h, 1203.12±389.50 vs 1321.41±333.52/1341.00±248.89/1563.57±51.01/1503.66±578.19 pg/mL, *P*_baseline vs post-PCI 3 h_ > 0.05, *P*_baseline vs post-PCI 6 h_ > 0.05, *P*_baseline vs post-PCI 12 h_ > 0.05, and *P*_baseline vs post-PCI 24 h_ > 0.05), (baseline vs non-PCI 3 h/non-PCI 6 h/non-PCI 12 h/non-PCI 24 h, 1512.13±313.83 vs 1656.39±479.47/1688.04±501.95/1639.56±556.17/ 1810.67±546.88 pg/mL, *P*_baseline vs non-PCI 3 h_ > 0.05, *P*_baseline vs non-PCI 6 h_ > 0.05, *P*_baseline vs non-PCI 12 h_ > 0.05, and *P*_baseline vs non-PCI 24 h_ > 0.05) (***Fig. 2C***). The level of early EPCs (%) was proportionally correlated with the plasma VEGF (pg/mL) (*r* = 0.48, *P* < 0.01) (***Fig. 3***).

## DISCUSSION

Cardiovascular diseases are the leading cause of death worldwide and corresponding risk assessment has become mandatory in the adult population[27]. Over the past decade, it has become clear that patients with a given combination of risk factors clustered in the so-called metabolic syndromes are more prone to CAD[28]. Apart from classic risk factors, novel biomarkers have recently received much attention[29] and an extreme plasticity is being attributed to apelin and EPCs.

In the present study, we found that the levels of apelin were lower in the AMI group than the CAD group and the control. This result suggested that apelin may be involved in the pathophysiological process of coronary artery stenosis. which is consistent with the findings of a previous study in which apelin may be a new plasma marker for cardiovascular disease in vivo[30],[31]. In this study, the findings also revealed increased level of early EPCs in AMI patients, which means enhanced mobilization of EPCs after AMI. This result agrees with the findings of EPCs participating in angiogenesis and reendothelialization after vascular injury[1],[5]−[7].

Moreover, correlation analysis showed a significant relationship between plasma apelin level, Gensini score and early EPC level. The higher Gensini score is accompanied by a lower apelin level. Gensini score is a useful tool to estimate both the extent and the severity of the lesions of coronary arteries in CAD[32]. So our result suggested that apelin was associated with the extent of coronary stenosis[33],[34]. This finding is consistent with previous reports that the apelin/APJ system played certain pathophysiological roles in vascular disease[5],[35], and apelin and APJ together may promote the formation of new blood vessels[36],[37]. The mechanisms for the asosication of reduced apelin levels with the severity of coronary artery stenosis have not been clarified. The biological roles of apelin are only beginning to be explored. An important view is that apelin directly activates the vascular L-Arg/NOS/NO pathway, which could be one of the most important mechanisms of apelin-regulated vascular function in a physiological condition[38].

EPCs in peripheral blood are thought to participate in postnatal vasculogenesis and ongoing repair of vascular endothelium. Enumeration and functional assessment of EPCs has been considered a novel technique for the assessment of vascular damage and reparative capacity. Elevated levels of early EPCs in peripheral blood may represent the ability of the human body to repair endothelial injuries. Early EPCs possess the capacity to home to sites of vascular injury, to perform neoangiogenesis in vivo and to maintain the homeostasis of vascular endothelium[8]. The level of early EPCs is also related to the degree of endothelial damage and coronary stenosis. The angiogenic activity of apelin may result from the combination of the molecule to APJ, playing a role in the proliferation, migration, and tube formation of endothelial cells[39]. EPCs are circulating angioblasts derived from human bone marrow with a potential to differentiate into endothelial cells[1],[2]. Therefore, our result suggested that apelin may be related to the level of early EPCs. This finding is consistent with previous reports that the apelin/APJ system played certain pathophysiological roles in vascular disease. Apelin activates cell transduction cascades such as serine/ threonine kinase (Akt) and extracellular signal-regulated kinases (ERKs)[23],[40], which lead to the proliferation of endothelial cells and the formation of new blood vessels[2]. Interestingly, knocking out of the apelin gene is associated with a delay in the development of retinal vasculature[41].

The strength of our findings is reflected by the precise methodology for the evaluation of EPCs. In the present study, cells analyzed by the flow cytometry analysis were "true" early EPCs, characterized as CD45^low/−^CD34^+^ KDR^+^CD133^+^ cells, rather than undefined progenitor cells[42],[43]. The level of EPCs measured by flow cytometry was previously suggested as a biomarker to assess cardiovascular disease risk[44],[45]. It is now accepted that EPCs may exert an important function as an endogenous repair mechanism to maintain the integrity of the endothelial monolayer by replacing lost cells in the denuded part of the artery[46]. Thus, the greater the number of early EPCs is, the better mobilization of EPCs from the bone marrow is, which may enhance more therapeutic properties.

This study provides new insights into the relationships between change in cytokines and mobilizations of EPCs by showing the change patterns of cytokines and early EPCs during the first 24 h after admission. In this study population, this association may be ascribed to the extent of vascular tree under ischemia, which is particularly large in this clinical condition and may represent a continuous stimulus for EPC mobilization from the bone marrow into peripheral blood. High VEGF levels were significantly associated with increased early EPC levels, suggesting that this cytokine may contribute to EPC mobilization. Two studies that support this result were conducted by Scheubel et al.[47] and Asahara et al.[48] who demonstrated a significant association between circulating VEGF and EPCs. Furthermore, our data were different from those obtained by Roberts et al.[49] and Francesca et al.[50], who failed to demonstrate a correlation between EPCs and VEGF after cardiac surgery, and by Shomig et al. in AMI patients, in whom high levels of IL-8, but not of VEGF, were associated with progenitor mobilization[11]. These may be related to different defining characteristics of target progenitor cells, different types of progenitor cell analysis as well as different methods used for determining VEGF and IL-8 levels.

Local delivery of SDF-1 can enhance EPC recruitment and neovascularization. Combined delivery of SDF-1 and EPCs into sites of limb ischemia promotes local EPC-mediated vasculogenesis. SDF-1 has also been shown to promote bone marrow cell proliferation and angiogenesis[51]. Although effects of SDF-1 have been relatively extensively studied in vitro and in animal models, the role of SDF-1 in humans and especially in patients with myocardial ischemia has been poorly described so far. The present findings fail to show enhanced plasma SDF-1 in the cases of acute tissue ischemia. This study included a small number of patients, and therefore the ability to generalize the differences was limited. Larger study population would provide higher statistical power. Besides, local other than plasma SDF-1 may contribute to enhance progenitor cell recruitment and neovascularization. Therefore, although the exact clinical significance of SDF-1 in patients with AMI remains to be elucidated, it is tempting to speculate that SDF-1 may play a role in vascular and myocardial remodeling or regeneration in patients with AMI. Further studies are needed to elucidate this pathophysiological mechanism. Understanding the role of VEGF, SDF-1 and apelin in EPCs function and tissue regeneration may help to develop novel therapeutic strategies in treatment of patients with myocardial damage.

This study indicated the possible correlation of plasma apelin levels with the severity of coronary artery stenosis and early EPCs in humans, and plasma VEGF with early EPCs. Endogenous apelin and EPCs are required for the repair of endothelial damage, and apelin[52] and early EPC induction together with VEGF significantly restore ischemic damages by formation of new blood vessels. From the pathophysiological point of view, it might be speculated that apelin acts as a vasopressor in damaged vasculature (e.g., atherosclerosis) and plasma apelin levels may be a useful indicator for the severity of coronary artery stenosis. The measurement of plasma apelin levels may be useful for predicting the severity of coronary artery stenosis.
